# Gait characteristics associated with the foot and ankle in inflammatory arthritis: a systematic review and meta-analysis

**DOI:** 10.1186/s12891-015-0596-0

**Published:** 2015-06-05

**Authors:** Matthew Carroll, Priya Parmar, Nicola Dalbeth, Mark Boocock, Keith Rome

**Affiliations:** 1 Department of Podiatry, Health & Rehabilitation Research Institute, Auckland University of Technology, Auckland, New Zealand; 2 National Institute for Stroke and Applied Neurosciences, Auckland University of Technology, Auckland, New Zealand; 3 Department of Medicine, University of Auckland, Auckland, New Zealand; 4 Department of Physiotherapy, Health & Rehabilitation Research Institute, Auckland University of Technology, Auckland, New Zealand

**Keywords:** Gait, Rheumatoid arthritis, Ankylosing spondylitis, Psoriatic arthritis, Gout

## Abstract

**Background:**

Gait analysis is increasingly being used to characterise dysfunction of the lower limb and foot in people with inflammatory arthritis (IA). The aim of the systematic review was to evaluate the spatiotemporal, foot and ankle kinematic, kinetic, peak plantar pressure and muscle activity parameters between patients with inflammatory arthritis and healthy controls.

**Methods:**

An electronic literature search was performed on Medline, CINAHL, SportsDiscus and The Cochrane Library. Methodological quality was assessed using a modified Quality Index. Effect sizes with 95 % confidence intervals (CI) were calculated as the standardised mean difference (SMD). Meta-analysis was conducted if studies were homogenous.

**Results:**

Thirty six studies with quality ranging from high to low met the inclusion criteria. The majority of studies reported gait parameters in Rheumatoid arthritis (RA). The gait pattern in RA was characterised by decreased walking speed (SMD 95 % CI −1.57, −2.25 to −0.89), decreased cadence (SMD −0.97, −1.49 to −0.45), decreased stride length (SMD −1.66, −1.84 to −1.49), decreased ankle power (SMD −1.36, −1.70 to −1.02), increased double limb support time (SMD 1.03, 0.84 to 1.22), and peak plantar pressures at the forefoot (SMD 1.11, 0.76 to 1.45). Walking velocity was reduced in psoriatic arthritis and gout with no differences in ankylosing spondylitis. No studies have been conducted in polymyalgia rheumatica, systemic sclerosis or systemic lupus erythematosus.

**Conclusions:**

The review identified the majority of studies reporting gait adaptations in RA, but limited evidence relating to other IA conditions. Poor data reporting, small sample sizes and heterogeneity across IA conditions limit the interpretation of the findings. Future studies may consider a standardised analytical approach to gait analysis that will provide clinicians and researchers with objective evidence of foot function in people with IA.

**Electronic supplementary material:**

The online version of this article (doi:10.1186/s12891-015-0596-0) contains supplementary material, which is available to authorized users.

## Background

The term ‘inflammatory arthritis’ (IA) has been used to describe a number of inflammatory joint diseases including: rheumatoid arthritis (RA), ankylosing spondylitis (AS), psoriatic arthritis (PsA) and gout [1]. RA is a chronic progressive autoimmune disease characterized by joint swelling, joint tenderness and destruction of synovial joints [2]. SpA encompasses a heterogeneous group of inflammatory arthritic conditions, characterised by vertebral involvement, peripheral oligoarthritis or polyarthritis, enthesitis, AS, PsA and undifferentiated spondyloenthesoarthritis [3, 4]. Gout is a common form of inflammatory arthritis caused by the deposition of monosodium urate crystals within joints and other soft tissue associated with hyperuricaemia [5]. IA causes lower limb and foot pain and impairment, functional disability, reduced mobility, joint deformity and altered gait strategy [6–10]. Foot pain is considered an important factor in the development of antalgic gait in IA, specifically in RA and gout [6, 11, 12]. In RA, foot pain is derived from structural and functional alterations associated with inflammatory and structural change [6, 13]. With the development of an antalgic gait, adaptations occur based upon a pain avoidance strategy. Previous studies have reported gait adaptations in RA and these include: a decrease in walking velocity and subsequent alterations to velocity related spatiotemporal parameters including, reduced cadence, increased double limb support time and decreased step length [14–18]. Changes to kinematic parameters including, reduced sagittal plane ankle ROM and increased peak rearfoot eversion have also been reported [7, 14, 17, 18]. Furthermore, previous studies have reported alteration to kinetic parameters including, reduced peak ankle plantarflexor power associated with reduced walking velocity, reduced ankle joint ROM, reduced ankle joint angular velocity, reduced ankle plantarflexor moments and decreased strength of the ankle plantarflexor muscles [16, 17, 19]. An increase in peak forefoot plantar pressure parameters has also been reported in RA [16].

Gait analysis provides information about spatial-temporal parameters, kinetics, kinematics and muscle activity to further delineate the relationship between joint disease, joint impairments and compensatory gait strategies adopted to overcome painful and disabling deformities [15, 20]. Gait analysis has been reported as a useful clinical tool to quantify foot function in both early and established RA [7, 8, 14, 15]. However, less common IA conditions, such as AS, PsA, gout, polymyalgia rheumatica, systemic sclerosis and systemic lupus erythematosus, also have various consequences for the lower limb such as changes in foot function, and extra articular complications involving the skin and vascular integrity [9, 10, 12, 21–24]. A recent systematic review of studies investigating walking abnormalities associated with RA, Baan [25] identified changes in gait such as a slower walking, longer double support time, and avoidance of extreme positions. These changes were in relation to the frequently found static features in RA, for instance, hallux valgus, pes planovalgus and rearfoot abnormalities. However, Baan [25] only reported gait parameters in RA and did not consider other IA conditions. However, recently there has been an interest in evaluating gait patterns in other IA conditions that includes gout [12], PsA [21] and AS [10]. No previous systematic review has conducted meta-analysis of gait parameters in IA compared to healthy control population. The aim of the systematic review was to evaluate spatiotemporal, foot and ankle kinematic, kinetic, peak plantar pressure and muscle activity parameters in people with IA and healthy controls.

## Methods

### Identification of studies

Four electronic databases were searched (Medline, CINAHL, SportsDiscus and The Cochrane Library). The search was completed in March 2015. The search strategy combined terms appropriate to the anatomical location; the type of gait analysis and IA condition (Additional file 1: Table S1). An initial review was undertaken of all titles and abstracts. All articles considered appropriate were read in full to establish if they met the eligibility criteria.

### Inclusion and exclusion criteria

Studies were included if they: reported people with IA that included; RA, AS, PsA, gout, polymyalgia rheumatica, systemic sclerosis and systemic lupus erythematosus; if they assessed adults aged >18 years old; if they reported spatiotemporal, kinematic, kinetic, peak plantar pressure or muscle activity data during gait; if they were articles that included a healthy group as means of comparison. Only articles published in English were included. Surgical and pharmacological intervention studies were excluded. No limitation was placed on the date of the publication with databases screened up to March 2015.

### Data extraction

All titles and abstracts identified through database searches were downloaded into Endnote X4 (Thomson, Reuters, Carlsbad, CA). Each title and abstract was evaluated for potential inclusion by two independent reviewers (MC, KR). If there was insufficient information contained in the title to determine suitability the full text was obtained. Any discrepancies between the two reviewers (MC, KR) were resolved at a consensus meeting.

### Assessment of methodological quality and diversity

The quality of studies was evaluated independently by two reviewers (MC and KR), who were blinded to author and publication details. Study quality was rated using a modified version of the Quality Index (QI) tool originally described by Downs and Black [26]. The QI tool consists of 27 items which allow for the assessment of internal and external validity, reporting of bias and power. The tool was modified to exclude thirteen questions that were not relevant to the articles assessed in this review, resulting in the retention of 14 questions. The scoring system grades each of the 14 questions either a (0 = no/unable to determine, or 1 = yes) with the exception of question five (0 = no, 1 = partially, 2 = yes). The summed score for each study was calculated, the maximum achievable being 15. No cut off scores have been described to categorise study quality for the Downs and Black quality Index [27]. In the absence of validated cut off scores and following review of past articles that have applied the Downs and Black criteria the follow cut off values were applied: ≥ 12 was considered high quality, ≥ 7 but < 12 as moderate quality, and < 6 as poor quality [27, 28].

### Data analysis and synthesis

Relevant gait parameters and information regarding overall study design, subject characteristics and gait analysis parameters were extracted from each paper by one author (MC) from those studies meeting the inclusion criteria. Data was tabulated according to the specific IA condition and gait parameters.

The clinical and methodological diversity among the studies was assessed to determine the appropriateness of data pooling for meta-analysis. Factors considered important for comparison included: mean age, sex distribution, case and comparison group size, data acquisition methodology and instrumentation. Two authors (MC and PP) reviewed the included studies and reached consensus on the appropriateness of conducting meta-analysis. Heterogeneity was considered low if the I^2^ value was ≤ 25 %, moderate if the value was > 25 % and ≤ 50 %, high if > 50 % and ≤ 75 % and very high if greater than 75 % [29]. A fixed-effect model was applied where the I^2^ statistic was less than 50 % and the Chi^2^ test indicated a non-significant degree of heterogeneity (*P* > 0.1). The random-effect model was used where the I^2^ statistic was greater than 50 % and the Chi^2^ test indicated statistically significant heterogeneity (*P* < 0.1) [30].

Where data was available from each paper a standardised mean difference (SMD) (Hedges’s g) and 95 % confidence interval (CI) were calculated [31]. This was calculated as the difference between cases and control group means divided by the pooled SD. Interpretation of SMDs was based on previous effect size (ES) guidelines: small effect ≥ 0.2, medium effect ≥ 0.5, large effect ≥ 0.8 [32]. Effect sizes were considered statistically significant if the 95 % CI did not contain zero for the SMD. All data were analysed using the Comprehensive Meta-analysis, version 2 [33]. When mean and SD was not reported, the median and range were reported. Studies that met the inclusion criteria but did not report SD, or where the SD could not be obtained were excluded from meta-analysis (Additional file 2: Table S2).

## Results

### Selection and characteristics of studies

**Fig. 1:**
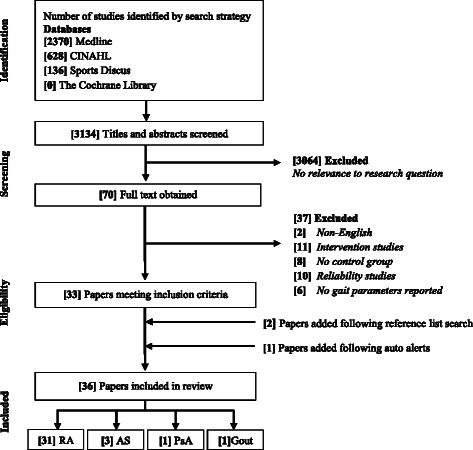
Flow of information through different stages of systematic review. RA = rheumatoid arthritis, AS = ankylosing spondylitis, PsA = psoriatic arthritis

A total of 3134 citations were identified for screening with 36 articles being included for further analysis (Fig. 1). Thirty-one studies evaluated gait parameters in RA [7, 8, 11, 13–18, 34–55], three in AS [10, 56, 57] one in PsA [21] and one in gout [12]. Twenty-four studies examined spatiotemporal gait parameters, with 19 in RA, two in AS, one PsA and gout (Additional file 3: Table S3). Twenty-one studies assessed kinematic parameters, with 17 in RA, three AS and one in PsA (Additional file 4: Table S4). Ten studies examined kinetic parameters with eight in RA, one AS and one in PsA (Additional file 5: Table S5). Sixteen studies evaluated plantar pressure parameters, with 15 in RA and one in gout (Additional file 6: Table S6). Three studies assessed all gait parameters (spatiotemporal, kinematic, kinetic and plantar pressures) in the population of interest [11, 14, 15]. No studies reported gait characteristics in polymyalgia rheumatica, systemic sclerosis and systemic lupus erythematosus. The total number of participants was 2275; 1321 with IA and 954 controls. IA participants included 863 females and 312 males. The mean (SD) age of IA cases and controls was 52.6 (9.3) and 47.8 (9.2) years, respectively (Table 1).

**Table 1:** Characteristics of included studies

Author	IA	Case demographics			Control demographics			Gait parameters investigated				
		Number	Gender (F:M)	Age	Number	Gender	Age	ST	KIM	KIN	PP	EMG
				mean (SD)		(F:M)	mean (SD)					
Turner [7]	RA	23	14:9	49.4 (10.5)	23	14:9	49.5 (13.6)	■	■		■	
Woodburn [8]	RA	11	9:2	59.6 (12.0)	5	NR	NR	■	■			
O’Connell [11]	RA	10	8:2	54.0	7	5:2	34.0^a^	■	■	■	■	
Woodburn [13]	RA	50	34:16	54.0 (11.8)	45	29:16	51.8 (12.4)		■			
Turner [14]	RA	12 (FF)	9:3	7.9 (9.3)	53	33:20	55.2 (11.7)	■	■		■	
		10 (RF)	8:2	53.8 (13.2)								
		6 (COMB)	4:2	64.7 (6.9)								
Turner [15]	RA	74	58:16	56.4 (12.0)	53	33:20	55.2 (11.7)	■	■	■	■	
Turner [16]	RA	12	12:0	46.0^a^	12	12:0	47.0^a^	■	■	■	■	
Weiss [17]	RA	50	43:7	55.0 (14.0)	37	22:15	51.0 (14.0)	■	■	■		
Khazzam [18]	RA	22	20:2	54.0^a^	25	12:13	41.0^a^	■	■			
Barn [19]	RA	10	6:4	50.0 (9.0)	5	3:2	47.0 (6.0)		■	■		■
Woodburn [34]	RA	10	NR	52.3^a^	10	NR	27.9^a^		■			
Bowen [36]	RA	114	93:21	59.6 (12.0)	49	37:12	33.2^a^				■	
Dubbeldam [37]	RA	21	17:4	46.6 (12.8)	14	11:3	41.6 (8.5)	■	■			
Yavuz [38]	RA	9	8:1	53.2 (12.3)	14	9:5	53.6 (18.7)				■	
Rome [39]	RA	19	15:4	56.1 (11.1)	21	12:9	51.0 (8.9)	■				
Eppeland [40]	RA	17	7:10	51.1 (6.2)	20	8:12	50.4 (5.3)	■				
Schmiegel [41]	RA	21	NR	57.1 (10.2)	16	NR	50.8 (9.4)				■	
Schmiegel [42]	RA	112	NR	55.0 (11.0)	20	NR	53.2 (12.3)				■	
Laroche [43]	RA	9	6:3	60.0 (7.0)	9	7:2	60.0 (7.0)	■	■			
Laroche [44]	RA	9	6:3	60.6 (6.8)	7	5:2	58.5 (7.4)	■	■	■		
Semple [45]	RA	74	58:16	54.6 (12.0)	53	33:20	55.2 (11.7)	■			■	
Rosenbaum [46]	RA	25	23:2	55.0 (9.9)	21	20:1	50.8 (9.3)				■	
Tuna [47]	RA	50	38:12	50.0 (9.0)	50	39:11	49.8 (7.6)				■	
Otter [48]	RA	25	21:4	45.3 (12.7)	25	22:3	48.0 (8.6)				■	
Woodburn [49]	RA	102	76:26	63.5^a^	42	31:11	61.0^a^				■	
Siegel [50]	RA	4	3:1	56.5 (7.2)	2	2:0	28.0 (11.0)		■	■		
Fransen [51]	RA	113	76:37	60.0 (5.5)	102	67:35	58.7 (5.3)	■				
Isacson [52]	RA	17	17:0	40.0 (5.0)	11	11:0	29.0 (7.0)	■	■			
Minns [53]	RA	124	104:20	56.6^a^	67	32:35	50.2 (10.2)	■			■	
Simkin [54]	RA	18	11:7	58.0^a^	20	10:10	51.0^a^	■		■		
Stauffer [55]	RA	30	18:12	NR	29	15:14	NR	■	■			
Del Din [10]	AS	12	4:8	49.4 (10.5)	12	4:8	55.75 (3.2)	■	■	■		
Mangone [56]	AS	17	2:15	47.0 (21.9)	10	1:9	38.7 (14.5)	■	■			
Zebouni [57]	AS	12	4:8	46.5^a^	11	NR	39.5^a^	■	■			
Woodburn [21]	PsA	42	25:17	45.3 (12.7)	29	18:11	40.0 (10.5)	■	■	■		
Rome [12]	GT	25	6:19	61.2 (11.7)	25	6:19	57.3 (12.2)	■			■	

*SD* standard deviation, *NR* not reported, *IA* inflammatory arthritis, *RA* rheumatoid arthritis, *GT* gout, *AS* ankylosing spondylitis, *PsA* psoriatic arthritis, *ST* spatiotemporal, *KIM* kinematic, *KIN* kinetic, *PP* plantar pressure, *EMG* electromyography, *FF group* severe forefoot deformity group, *RF group* severe rearfoot deformity group, *COMB group* severe fore- and rearfoot deformity group

^a^ SD not reported

### Methodological quality of studies

Two reviewers (MC & KR) individually scored a total of 504 items and agreed on 480 items (95 %) with an inter-rater agreement of ƙ = 0.90 (*p* < 0.001). Six of the 36 articles were of high quality (quality score ≥ 12). The median (%) quality score of all articles was 10 (67 %), ranging between 20–87 % (Table 2). There was limited information on the methods of study recruitment across the majority of studies making it difficult to assess the generalisability of study results. The majority of studies investigating kinematic and kinetic parameters also reported small sample sizes.

**Table 2:** Modified quality index

Publication	Reporting							External validity		Internal validity bias			Internal validity confounding		
	1. Hypothesis clearly described?	2. Main outcomes clearly described?	3. Characteristics of the patients included clearly described?	5. Distribution of principle confounder of each group clearly described?	6. Main findings clearly described?	7. Estimates of random variability provided for the main outcomes?	10. Actual probability values reported for main outcomes?	11. Were the subjects asked to participate representative of the entire population?	12. Were the subjects who were prepared to participate representative of the entire population?	16. Was it clear if the results were based on “data dredging”	18. Were the statistical tests appropriate?	20. Were the main outcome measures valid and reliable?	21. Were all patients and controls recruited from the same population?	22. Were all patients and controls recruited over the same time period?	Quality Index score total (%)
Turner [7]	1	1	1	1	1	1	0	0	0	1	1	0	0	0	8 (53)
Woodburn [8]	1	1	0	0	0	1	0	0	0	1	0	1	0	0	5 (33)
Del Din [10]	1	1	1	2	1	1	1	1	0	1	1	1	1	0	13 (87)
O’Connell [11]	1	1	0	0	1	0	0	0	0	1	0	0	0	0	4 (27)
Rome [12]	1	1	1	1	1	1	1	0	0	1	1	1	1	0	11 (73)
Woodburn [13]	1	1	1	2	1	1	1	0	0	1	1	1	0	0	11 (73)
Turner [14]	1	1	1	2	1	1	1	0	0	1	1	1	1	0	12 (80)
Turner [15]	1	1	1	2	1	1	0	0	0	1	1	1	0	0	10 (67)
Turner [16]	1	1	1	1	1	0	0	1	1	1	0	1	1	0	10 (67)
Weiss [17]	1	1	0	1	1	1	1	0	0	1	1	1	1	0	10 (67)
Khazzam [18]	1	1	0	0	1	1	1	1	0	1	1	1	1	1	11 (73)
Barn [19]	1	1	1	2	1	1	1	0	0	1	1	0	0	1	11 (73)
Woodburn [21]	1	1	1	2	1	1	1	0	0	1	1	0	0	0	10 (67)
Woodburn [34]	1	1	1	2	1	0	0	1	0	1	1	1	1	0	11 (73)
Bowen [36]	1	1	1	2	1	1	1	0	0	1	1	1	0	1	12 (80)
Dubbeldam [37]	1	1	1	1	0	1	1	0	0	1	1	1	0	0	9 (60)
Yavuz [38]	1	1	0	0	0	1	0	0	0	1	1	0	0	0	5 (33)
Rome [39]	1	1	1	1	1	1	1	0	0	1	1	0	0	0	9 (60)
Eppeland [40]	1	1	1	2	1	1	1	1	1	1	1	0	1	0	13 (87)
Schmiegel [41]	1	1	1	2	1	1	0	0	0	1	1	1	0	0	10 (67)
Schmiegel [42]	1	1	1	2	1	1	0	1	1	1	1	0	1	0	12 (80)
Laroche [43]	1	1	1	0	1	0	1	0	0	1	0	0	0	0	6 (40)
Laroche [44]	1	1	1	0	1	1	1	1	1	1	1	1	0	0	11 (73)
Semple [45]	1	1	1	0	1	1	1	1	1	1	1	1	0	0	11 (73)
Rosenbaum [46]	1	1	1	1	1	1	1	0	0	1	1	1	0	0	10 (67)
Tuna [47]	1	1	1	1	1	1	0	0	0	1	1	0	0	0	8 (53)
Otter [48]	1	0	1	1	0	1	1	0	0	1	1	0	1	0	8 (53)
Woodburn [49]	1	1	1	2	1	1	1	0	0	1	1	1	0	0	11 (73)
Siegel [50]	1	1	0	0	0	0	0	0	0	1	0	0	0	0	3 (20)
Fransen [51]	1	1	1	2	1	1	1	0	1	1	1	0	1	0	12 (80)
Isacson [52]	1	0	1	0	1	0	0	0	0	1	1	0	1	0	6 (40)
Minns [53]	0	0	1	1	1	0	0	0	0	1	0	0	0	0	4 (27)
Simkin [54]	1	1	0	0	0	0	0	0	0	1	0	0	0	0	3 (20)
Stauffer [55]	1	1	1	1	1	0	0	0	0	1	0	1	0	1	8 (53)
Mangone [56]	1	1	1	0	0	1	1	0	0	1	1	0	0	0	7 (47)
Zebouni [57]	1	1	0	0	0	0	0	0	0	1	1	0	0	0	4 (27)
													Median		10 (67)

### Spatiotemporal gait parameters

**Fig. 2:**
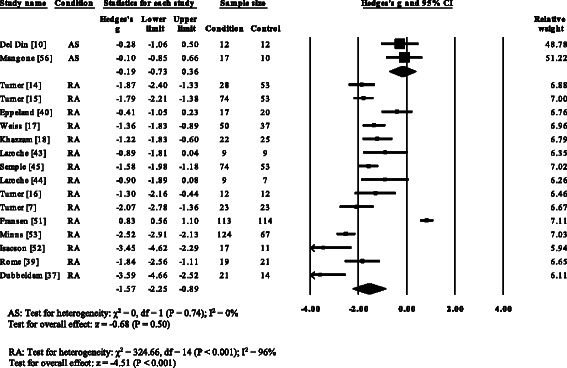
Forest plot of studies reporting walking velocity. AS, ankylosing spondylitis; RA, rheumatoid arthritis; CI, confidence interval

Fifteen RA [7, 14–18, 37, 39, 40, 44, 45, 51–54], one PsA [21] and one gout study [12] reported significant decreases in walking velocity. No significant differences in walking velocity were reported for AS [10, 56]. Overall pooled data (SMD, 95 % CI) (Fig. 2) for walking velocity, demonstrated a significant decreased large effect size for RA (SMD −1.55, −2.27 to −0.83) and a non-significant decrease for AS (SMD −0.19, −0.73 to 0.36).

**Fig. 3:**
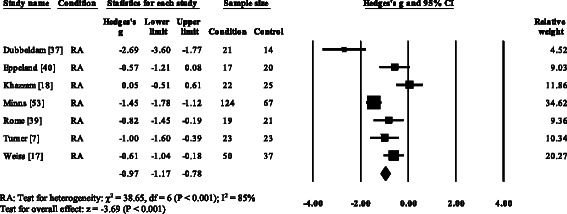
Forest plot of studies reporting cadence. RA, rheumatoid arthritis; CI, confidence interval

**Fig. 4:**
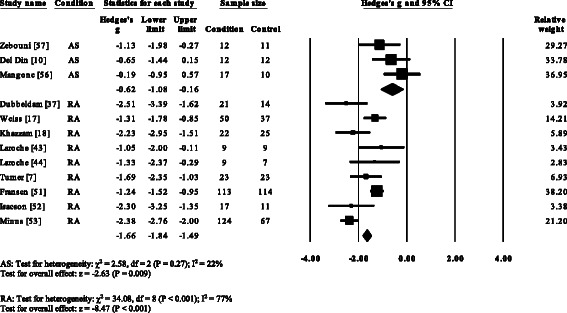
Forest plot of studies reporting stride length. AS, ankylosing spondylitis; RA, rheumatoid arthritis; CI, confidence interval

**Fig. 5:**
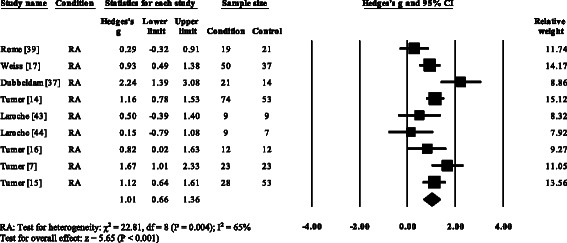
Forest plot of studies reporting double support time. RA, rheumatoid arthritis; CI, confidence interval

Five RA studies [7, 17, 37, 39, 53] and one gout (52) reported significant decreases in cadence. Cadence was not significantly decreased in AS [57]. Overall, pooled data for cadence in RA (Fig. 3) showed a decreased but significant large effect size (SMD −0.97, −1.49 to −0.45). Nine RA studies [7, 17, 18, 37, 44, 51–54], one AS [57] and one gout [12] reported significant decreases in stride length. Pooled data for stride length in RA (SMD −1.66, −1.84 to −1.49) and AS (SMD −0.62, −1.08 to −0.27) were significantly decreased with a large effect size (Fig. 4). Eight RA studies [7, 14–17, 37, 39, 51] and one gout study [12] reported significant increases in double support. Pooled data for double support in RA showed (Fig. 5) a significantly increased large effect size (SMD 1.01, 0.66 to 1.36).

### Kinematic and kinetic gait parameters

**Fig. 6:**
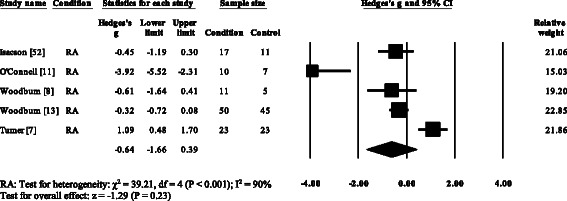
Forest plot of studies reporting ankle range of motion. RA, rheumatoid arthritis; CI, confidence interval

**Fig. 7:**
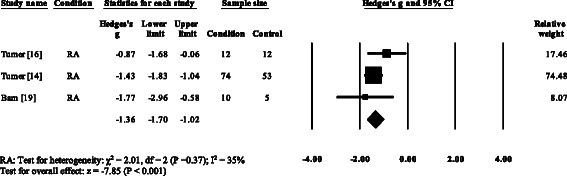
Forest plot of studies reporting ankle power. RA, rheumatoid arthritis; CI, confidence interval

Five RA studies reported on the total ankle range of motion [7, 8, 11, 13, 52]. Three studies reported no significant differences [8, 13, 52], with one study reporting a significant increase [7] and one study reporting a significant decrease in the total ankle range of motion [11]. Results of the meta-analysis (Fig. 6) demonstrated that the overall effect size for total ankle range of motion was non-significant (SMD −0.64, −1.66 to 0.39). Ankle power was reported in three RA [15, 16, 35] and one PsA study [21]. All four studies reported significant reductions in ankle power. The overall effect size for ankle power in RA (Fig. 7) was significantly large (SMD −1.36, −1.70 to −1.02).

### Peak plantar pressure gait parameters

**Fig. 8:**
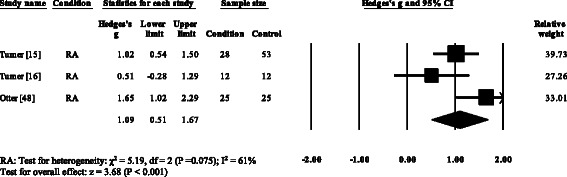
Forest plot of studies reporting forefoot peak plantar pressure. RA, rheumatoid arthritis; CI, confidence interval

**Fig. 9:**
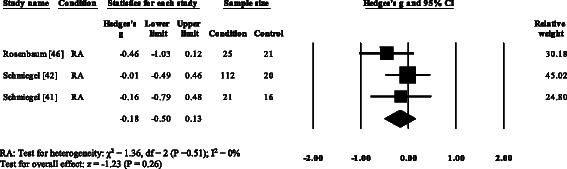
Forest plot of studies reporting rearfoot peak plantar pressure. RA, rheumatoid arthritis; CI, confidence interval

**Fig. 10:**
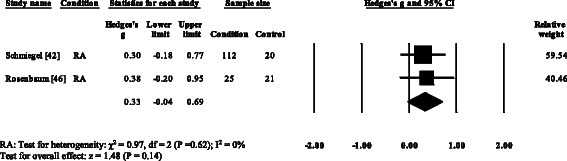
Forest plot of studies reporting midfoot peak plantar pressure. RA, rheumatoid arthritis; CI, confidence interval

**Fig. 11:**
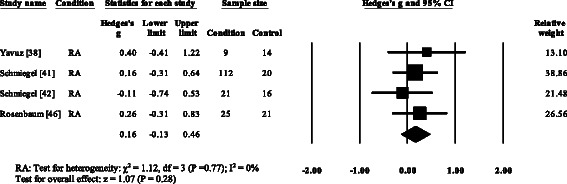
Forest plot of studies reporting 1^st^ metatarsophalangeal peak plantar pressure. RA, rheumatoid arthritis; CI, confidence interval

**Fig. 12:**
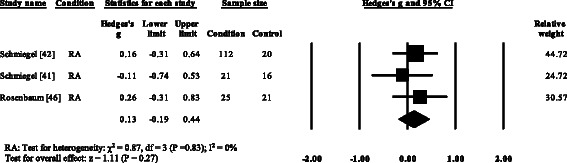
Forest plot of studies reporting 2^nd^ metatarsophalangeal peak plantar pressure. RA, rheumatoid arthritis; CI, confidence interval

**Fig. 13:**
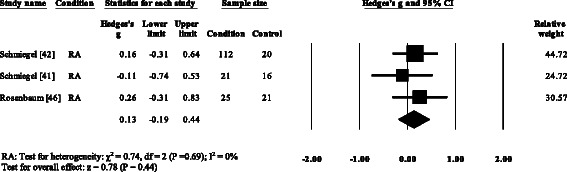
Forest plot of studies reporting 3^rd^ to 5^th^ metatarsophalangeal peak plantar pressure. RA, rheumatoid arthritis; CI, confidence interval

**Fig. 14:**
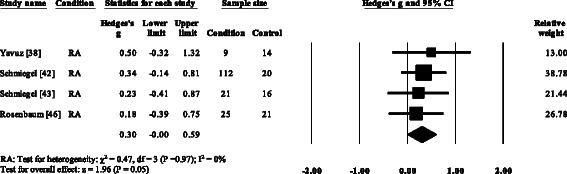
Forest plot of studies reporting hallux peak plantar pressure. RA, rheumatoid arthritis; CI, confidence interval

Three RA studies [14, 16, 47, 48] reported significantly higher forefoot peak plantar pressures in RA. Results from the meta-analysis (Fig. 8) showed that the overall effect size for peak plantar pressure to the forefoot was significantly large (SMD 1.09, 0.51 to 1.67). Pooled results in the RA studies demonstrated no significant differences in peak plantar pressure for the rearfoot (Fig. 9), midfoot (Fig. 10), first metatarsal (Fig. 11), 2^nd^ metatarsal (Fig. 12) and the 3–5^th^ metatarsal heads (Fig. 13). Hallux peak plantar pressure (Fig. 14) was reported to be significantly lower in gout [12].

### Muscle activity

One RA study investigated muscle activity of the tibialis posterior muscle and reported increased muscle activity during the single support phase of gait [35].

## Discussion

This systematic review highlights significant differences in gait variables between people with IA and controls. The review found the majority of studies report on RA with a limited number of studies on other IA conditions. The review found similar findings to previous studies, that people with RA adopt an antalgic gait resulting from a pain avoidance pattern that contributes to a decrease in walking velocity, cadence, increased double limb support time, and decreased ankle power with increased peak plantar pressures to the forefoot [11, 15, 17, 18]. Antalgic gait was also found in gout and AS suggesting that adaptation may occur due to the disease or a compensatory mechanism to accommodate for localised foot pain and deformity [14]. Gait adaptation in PsA may relate to entheseal foot pathologies and foot pain [9, 58]. Woodburn [21] postulated a stress shielding mechanism may be the driver of gait adaptation with walking speeds decreased in attempt to lower stress at the Achilles tendon. The review found a reduction in peak plantar pressure under the first metatarsal head, suggesting that people with gout may use a pain-avoidance strategy to reduce the pain associated with the structural joint damage of the first metatarsophalangeal joint.

The chief advantage of three-dimensional (3D) motion analysis is that dynamic assessments of foot motion during functional activities, such as walking, can be performed [59]. Recent advances in motion capture technology afford improved spatial resolution and allow the definition of relatively small segments in the foot [59]. In the last decade there has been an exponential growth in the use of 3D models to explain gait strategies [60]. The development of detailed foot models is beginning to quantify the kinematics and kinetics of the foot, however there are limitations for use in people with IA. Issues related to soft tissue artefacts and the validity of skin markers to track underlying skeletal segments remains problematic. Inaccurate identification of anatomical landmarks due to the presence of foot deformity in IA may affect the estimation, interpretation and reconstruction of joint axis and ultimately the calculation of joint kinematics and kinetics [61]. The development of foot models has also increased the detail and variety of 3D motion analysis variables used to explain gait strategies in people with IA. In comparison to spatiotemporal gait parameters and plantar pressure variables there appears to be no consensus as to the most important gait variables that relate to overall functional status.

This review has some limitations. There was a large variation in the disease activity, disease duration and level of deformities across all studies. Many studies used relatively small samples that were underpowered and the heterogeneous nature of the IA population makes interpretation of the data difficult. A number of studies were included in the review but excluded from data pooling due to a lack of data reporting of standard deviations and mean values of gait parameters. Previous studies have described a wide range of methodologies to acquire and define gait parameters and this complicates the synthesis of data across different studies. The review was restricted to case-control studies and did not consider findings from intervention studies. We only analysed the foot and ankle characteristics in IA, with no consideration given to data from the knee, hip and pelvis.

Two key pathways have been postulated to contribute to the development of foot pain and deformity in IA: inflammatory and/or mechanical [59]. However, limited objective evidence exists to comprehensively examine inflammatory and mechanical markers in the context of foot pain and deformity across IA conditions. Given the limited data across all IA conditions, future directions should include analysis of muscle activity; this will provide information on the forces producing movements and patterns of muscle activation. Future research is required to understand the combined effects of spatiotemporal, kinematic, kinetic and plantar pressure impact on foot function. This will allow for relationships to be investigated across the differing gait parameters and may further define the mechanism of gait adaptation with IA conditions.

## Conclusion

The advancement of 3D gait analysis has given a clearer insight into the complex interaction between the underlying mechanisms of inflammation and mechanical pathways that influence the development of foot problems in people with IA. The review identified 36 gait studies with the majority of studies reporting gait adaptations in RA, but limited evidence relating to other IA conditions. Poor data reporting, small sample sizes and heterogeneity across IA conditions limit the interpretation of the findings. Future studies should consider a standardised analytical approach to gait analysis that will enable comparisons across studies and provide clinicians and researchers with objective evidence of foot function in people with IA.

## Additional files

Additional file 1: **Table S1.** Description of search strategy. Details of specific search terms used and the order in which the search tool place.
Additional file 2: **Table S2.** Excluded studies. Details of the publications excluded, the reason for exclusion and the reference list of the excluded studies.
Additional file 3: **Table S3.** Spatiotemporal gait analysis parameters acquired by included studies. Details of the data acquisition method and the specific spatiotemporal parameters that were measured by studies assessing spatiotemporal gait parameters.
Additional file 4: **Table S4.** Kinematic gait parameters measured and methods of data acquisition. Details of the specific kinematic parameters, measured the biomechanical model used to determine kinematic parameters.
Additional file 5: **Table S5.** Kinetic gait analysis parameters acquired by included studies. Details of kinetic gait parameters measured.
Additional file 6: **Table S6.** Plantar pressure parameters acquired by included studies. Details of the specific regions of the foot where plantar pressure was measured and the method by which plantar pressure was acquired.
