# Supplementary material for: Gait characteristics associated with the foot and ankle in inflammatory arthritis: a systematic review and meta-analysis
Source: BMC Musculoskelet Disord. 2015 Jun 5;16:134. doi: 10.1186/s12891-015-0596-0 (PMC4455329; doi:10.1186/s12891-015-0596-0)
Supplement: Additional file 1: — Table S1. Description of search strategy. Details of specific search terms used and the order in which the search tool place. [file 12891_2015_596_MOESM1_ESM.pdf]

Gait characteristics associated with the foot and ankle in inflammatory arthritis: a systematic review and meta-analysis

Matthew Carroll, Priya Parmar, Nicola Dalbeth, Mark Boocock, Keith Rome

Additional 1. Description of search strategy.

Database-specific subject terms and truncated keywords were sorted into two distinct concepts: (a) gait analysis and (b) inflammatory rheumatic disease. Within each concept, subject terms were searched individually while keywords were combined with the term 'OR' into a single search. Subject terms and keywords within each concept were then combined with the term 'OR' to remove duplicates. Following this, the final search results for the two concepts will be combined together with the term 'AND' to form the total yield. This technique was applied to Medline (Additional Table 1) and CINAHL. SportDiscus and the Cochrane Library were searched by use of keywords only, as subject term functions are not available in these databases. Targeted searching of relevant journals also occurred following bibliographic review of retrieved articles.

Additional Table 1. Search strategy

**a**	1	Subject term	exp. Gait analysis
	2	Keywords	Lower extremi* [or] Lower limb* [or] Foot [or] Feet [or] Ankle* [or] Leg* [or] Rear foot [or] Hind foot [or] Knee [or] Hip
	3	Keywords	Kinematic* [or] Kinetic* [or] Spatial [or] Temporal [or] Spatio-temporal [or] electromy* [or] EMG [or] plantar pressure [or] pressure [or] force [or] pedobarogr*
	4	Combine	2 [and] 3
	5	Combine	1 [or] 4
**b**	6	Subject term	exp. Rheumatic disease
	7	Subject term	exp. Rheumatoid arthritis
	8	Subject term	exp. Gout
	9	Subject term	exp. Systemic sclerosis
	10	Subject term	exp. Polymyalgia rheumatica
	11	Subject term	exp. Psoriatic arthritis
	12	Subject term	exp. Lupus erythematosus
	13	Subject term	exp. Ankylosing spondylitis
	14	Keywords	Rheumat* [or] Spondyl*
**c**	15	Combine	6 [or] 7 [or] 8 [or] 9 [or] 10 [or] 11 [or] 12 [or] 13 [or] 14
	16	Combine	5 [and] 15

(a) Search terms for gait analysis (b) search terms for inflammatory rheumatic disease (c) combination of search terms
