# Supplementary material for: Gait characteristics associated with the foot and ankle in inflammatory arthritis: a systematic review and meta-analysis
Source: BMC Musculoskelet Disord. 2015 Jun 5;16:134. doi: 10.1186/s12891-015-0596-0 (PMC4455329; doi:10.1186/s12891-015-0596-0)
Supplement: Additional file 2: — Table S2. Excluded studies. Details of the publications excluded, the reason for exclusion and the reference list of the excluded studies. [file 12891_2015_596_MOESM2_ESM.pdf]

Gait characteristics associated with the foot and ankle in inflammatory arthritis: a systematic review and meta-analysis

Matthew Carroll, Priya Parmar, Nicola Dalbeth, Mark Boocock, Keith Rome

Additional File 2. Studies excluded from inclusion and meta-analysis

Publication		Grounds for exclusion inclusion in review	Grounds for exclusion from meta-analysis
Hyslop	[1]	Reliability study	
Sahin	[2]	No specific gait parameters reported	
Sakauchi	[3]	No specific gait parameters reported	
Sharma	[4]	No specific gait parameters reported	
Collis	[5]	No specific gait parameters reported	
Esbjörnsson	[6]	Dimensionless measurement units	
Barn	[7]	Reliability study	
Siddle	[8]	No healthy control group	
Sawacha	[9]	No healthy control group	
Dubbeldam	[10]	No healthy control group	
van der Leeden	[11]	No healthy control group	
Rome	[12]	Reliability study	
Hamilton	[13]	No healthy control group	
Fransen	[14]	Reliability study	
Keenan	[15]	No healthy control group	
Platto	[16]	No healthy control group	
Marshall	[17]	Control group no clear	
Kettelkamp	[18]	No healthy control group	
Brinkmann	[19]	Intervention	
Novak	[20]	Intervention	
Garling	[21]	Intervention	
Weiss	[22]	Intervention	
Mejjad	[23]	Intervention	
Valkeinen	[24]	Intervention	
Woodburn	[25]	Intervention	
Moffet	[26]	Intervention	
Meyers	[27]	Intervention	
Fransen	[28]	Intervention	
Locke	[29]	Intervention	
Tastekin	[30]	Non-English	
Miu	[31]	Non-English	
Doorn	[32]	Reliability study	
Brenton-Rule	[33]	Reliability study	
Vidmar	[34]	Reliability study	
Firth	[35]	Reliability study	
Eastlack	[36]	Reliability study	
Barrett	[37]	Reliability study	
Woodburn	[38]		No standard deviation value provided
O'Connell	[39]		No standard deviation value provided
Simkin	[40]		No standard deviation value provided
Stauffer	[41]		No standard deviation value provided
Woodburn	[42]		No standard deviation value provided
Turner	[43]		No standard deviation value provided
Tuna	[44]		Peak values reported not mean values
Woodburn	[45]		No standard deviation value provided

Additional File 2. Reference list

1. Hyslop E, Woodburn J, McInnes I, Semple R, Newcombe L, Hendry G, et al. A reliability study of biomechanical foot function in psoriatic arthritis based on a novel multi-segmented foot model. Gait Posture 2010;**32**:619-26.
2. Sahin N, Ozcan E, Baskent A, Karan A, Kasikcioglu E. Muscular kinetics and fatigue evaluation of knee using by isokinetic dynamometer in patients with ankylosing spondylitis. Acta Reumatol Port 2011;**36**:252-9.
3. Sakauchi M, Narushima K, Sone H, Kamimaki Y, Yamazaki Y, Kato S, et al. Kinematic approach to gait analysis in patients with rheumatoid arthritis involving the knee joint. Arthritis Care Res 2001;**45**:35-41.
4. Sharma M, Dhanendran M, Hutton WC, Corbett M. Changes in load bearing in the rheumatoid foot. Ann Rheum Dis 1979;**38**:549-52.
5. Collis W, Jayson M. Measurement of pedal pressures. An illustration of a method. Ann Rheum Dis 1972;**31**:215.
6. Broström EW, Esbjörnsson A-C, von Heideken J, Iversen MD. Gait deviations in individuals with inflammatory joint diseases and osteoarthritis and the usage of three-dimensional gait analysis. Best Pract Res Clin Rheumatol. 2012;**26**:409-22
7. Barn R, Rafferty D, Turner D, Woodburn J. Reliability study of tibialis posterior and selected leg muscle EMG and multi-segment foot kinematics in rheumatoid arthritis associated pes planovalgus. Gait Posture 2012;**36**:567-571.
8. Siddle HJ, Firth J, Waxman R, Nelson EA, Helliwell PS. A case series to describe the clinical characteristics of foot ulceration in patients with rheumatoid arthritis. Clin Rheumatol 2012;**31**:541-5.
9. Sawacha Z, Carraro E, Del Din S, Guiotto A, Bonaldo L, Punzi L, et al. Biomechanical assessment of balance and posture in subjects with ankylosing spondylitis. J Neuroeng Rehabil 2012;**9**:1-11.
10. Dubbeldam R, Baan H, Nene AV, Drossaers-Bakker KW, van de Laar MAFJ, Hermens HJ, et al. Foot and ankle kinematics in rheumatoid arthritis: The influence off foot and ankle joint and leg tendon pathologies. Arthritis Care Res 2012;65:503-511.
11. van der Leeden M, Steultjens M, Dekker JHM, Prins APA, Dekker J. Forefoot joint damage, pain and disability in rheumatoid arthritis patients with foot complaints: the role of plantar pressure and gait characteristics. Rheumatology 2006;**45**:465-9.
12. Rome K, Hanchard NCA. Within-day reliability of temporal-spatial gait parameters associated with rheumatoid arthritic feet. Musculoskeletal Care 2005;**3:17-**23.
13. Hamilton J, Brydson G, Fraser S, Grant M. Walking ability as a measure of treatment effect in early rheumatoid arthritis. Clin Rehabil 2001;**15**:142-7.
14. Fransen M, Edmonds J. Gait variables: appropriate objective outcome measures in rheumatoid arthritis. Rheumatology (Oxford) 1999;**38**:663-7.
15. Keenan M, Peabody T, Gronley J, Perry J. Valgus deformities of the feet and characteristics of gait in patients who have rheumatoid arthritis. J Bone Joint Surg Am 1991;**73**:237-47.
16. Platto MJ, O'Connell PG, Hicks JE, Gerber LH. The relationship of pain and deformity of the rheumatoid foot to gait and an index of functional ambulation. J Rheumatol. 1991;**18**:38-43.
17. Marshall RN, Myers DB, Palmer DG. Disturbance of gait due to rheumatoid disease. J Rheumatol 1980;**7**:617-23.
18. Kettelkamp D, Leaverton P, Misol S. Gait characteristics of the rheumatoid knee. Arch Surg 1972;**104**:30.
19. Brinkmann JR, Perry J. Rate and range of knee motion during ambulation in healthy and arthritic subjects. Phys Ther 1985;**65**:1055-60.
20. Novak P, Burger H, Tomsic M, Marincek C, Vidmar G. Influence of foot orthoses on plantar pressures, foot pain and walking ability of rheumatoid arthritis patients - a randomised controlled study. Disabil Rehabil 2009;**31**:638-45.

21. Garling E, Wolterbeek N, Velzeboer S, Nelissen R, Valstar E, Doorenbosch C, et al. Co-contraction in RA patients with a mobile bearing total knee prosthesis during a step-up task. Knee Surg Sports Traumatol Arthrosc 2008;**16**:734-40.
22. Weiss RJ, Broström E, Stark A, Wick MC, Wretenberg P. Ankle/hindfoot arthrodesis in rheumatoid arthritis improves kinematics and kinetics of the knee and hip: a prospective gait analysis study. Rheumatology 2007;**46**:1024-8.
23. Mejjad O, Vittecoq O, Pouplin S, Grassin-Delyle L, Weber J, Le Loet X. Foot orthotics decrease pain but do not improve gait in rheumatoid arthritis patients. Joint Bone Spine 2004;**71**:542-5.
24. Valkeinen H, Alen M, Hannonen P, Häkkinen A, Airaksinen O, Häkkinen K. Changes in knee extension and flexion force, EMG and functional capacity during strength training in older females with fibromyalgia and healthy controls. Rheumatology (Oxford) 2004;**43**:225-8.
25. Woodburn J, Helliwell PS, Barker S. Changes in 3D joint kinematics support the continuous use of orthoses in the management of painful rearfoot deformity in rheumatoid arthritis. J Rheumatol 2003;**30**:2356-64.
26. Moffet H, Noreau L, Parent E, Drolet M. Feasibility of an eight-week dance-based exercise program and its effects on locomotor ability of persons with functional class III rheumatoid arthritis. Arthritis Care Res 2000;**13**:100-11.
27. Meyers LL, Dobson SR, Wiegand D, Webb JD, Mencio GA. Mechanical instability as a cause of gait disturbance in high-grade spondylolisthesis: a pre- and postoperative three-dimensional gait analysis. J Pediatr Orthop 1999;**19**:672-6.
28. Fransen M, Edmonds J. Off-the-shelf orthopedic footwear for people with rheumatoid arthritis. Arthritis Care Res. 1997;10(4):250-6.
29. Locke M, Perry J, Campbell J, Thomas L. Ankle and Subtalar Motion During Gait in Arthritic Patients. Phys Ther 1984;**64**:504-9.
30. Tastekin N, Tuna H, Birtane M, Uzunca K. Plantar pressure changes of patients with heel valgus in rheumatoid arthritis. Turk J Rheumatol 2009;**24**:67-71.
31. Miu S, Ancuţa C, Belibou I, Macovei L, Chirieac R. [Disability assessment tools in psoriatic arthritis: VICON gait]. Rev Med Chir Soc Med Nat Iasi 2012;**116**:50-5.
32. Doorn PF, Keijsers NLW, van Limbeek J, Anderson PG, Laan RFJM, Bosch PVtP, et al. A clinical classification system for rheumatoid forefoot deformity. Foot Ankle Surg 2011;**17**:158-65.
33. Brenton-Rule A, Mattock J, Carroll M, Dalbeth N, Bassett S, Menz HB, et al. Reliability of the TekScan MatScan® system for the measurement of postural stability in older people with rheumatoid arthritis. J Foot Ankle Res 2012;**5**:1-7.
34. Vidmar G, Novak P. Reliability of in-shoe plantar pressure measurements in rheumatoid arthritis patients. Int J Rehabil Res 2009;**32**:36-40.
35. Firth J, Turner DE, Smith W, Helliwell PS, Woodburn J. Reliability of Pressurestat™ for measuring plantar foot pressures in patients with rheumatoid arthritis. Clin Biomechanics 2008;**23**:670-1.
36. Eastlack ME, Arvidson J, Snyder-Mackler L, Danoff JV, McGarvey CL. Interrater reliability of videotaped observational gait-analysis assessments. Phys Ther 1991;**71**:465-72.
37. Barrett JP, Jr. Plantar pressure measurements. Rational shoe-wear in patients with rheumatoid arthritis. JAMA 1976;**235**:1138-9.
38. Woodburn J, Nelson K, Siegel K, Kepple T, Gerber L. Multisegment foot motion during gait: proof of concept in rheumatoid arthritis. J Rheumatol 2004;**31**:1918-27.
39. O'Connell PG, Lohmann Siegel K, Kepple TM, Stanhope SJ, Gerber LH. Forefoot deformity, pain, and mobility in rheumatoid and nonarthritic subjects. J Rheumatol 1998;**25**:1681-6.
40. Simkin A. The dynamic vertical force distribution during level walking under normal and rheumatic feet. Rheumatol Rehabil 1981;**20**:88-97.

41. Stauffer RN, Chao EY, Györy AN. Biomechanical gait analysis of the diseased knee joint. Clin Orthop Relat Res. 1977(126):246-55.
42. Woodburn J, Turner D, Helliwell P, Barker S. A preliminary study determining the feasibility of electromagnetic tracking for kinematics at the ankle joint complex. Rheumatology (Oxford) 1999;**38**:1260-8.
43. Turner DE, Helliwell PS, Siegel KL, Woodburn J. Biomechanics of the foot in rheumatoid arthritis: Identifying abnormal function and the factors associated with localised disease 'impact'. Clin Biomech 2008;**23**:93-100.
44. Tuna H, Birtane M, Taştekin N, Kokino S. Pedobarography and its relation to radiologic erosion scores in rheumatoid arthritis. Rheumatol Int 2005;**26**:42-7.
45. Woodburn J, Helliwell PS. Relation between heel position and the distribution of forefoot plantar pressures and skin callosities in rheumatoid arthritis. Ann Rheum Dis 1996;**55**:806-10.
