# Supplementary material for: Gait characteristics associated with the foot and ankle in inflammatory arthritis: a systematic review and meta-analysis
Source: BMC Musculoskelet Disord. 2015 Jun 5;16:134. doi: 10.1186/s12891-015-0596-0 (PMC4455329; doi:10.1186/s12891-015-0596-0)
Supplement: Additional file 3: — Table S3. Spatiotemporal gait analysis parameters acquired by included studies. Details of the data acquisition method and the specific spatiotemporal parameters that were measured by studies assessing spatiotemporal gait parameters. [file 12891_2015_596_MOESM3_ESM.pdf]

Gait characteristics associated with the foot and ankle in inflammatory arthritis: a systematic review and meta-analysis

Matthew Carroll, Priya Parmar, Nicola Dalbeth, Mark Boocock, Keith Rome

Additional File 3: Spatiotemporal gait parameters measured and methods of data acquisition

Author & reference		IA	Data acquisition method	Parameters measured
Dubbeldam	(1)	RA	3D gait analysis	Walking velocity, cadence, stride time, double stance percentage, stride length, stride width, step length
Rome	(2)	RA	Electronic Walkway	Walking velocity, cadence, gait cycle time, single support time, double limb support time, base of support
Eppeland	(3)	RA	Electronic Walkway	Walking velocity, cadence, step length, stance phase duration, step width
Turner	(4)	RA	Electronic Walkway	Walking velocity, double support time
Turner	(5)	RA	Electronic Walkway	Walking velocity, double support time
Weiss	(6)	RA	3D gait analysis	Walking velocity, cadence, stride length, step length, single support, double support
Turner	(7)	RA	Electronic Walkway	Walking velocity, double support percentage
Turner	(8)	RA	Electronic Walkway	Walking velocity, cadence, gait cycle time, stride length, double support time
Laroche	(9)	RA	3D gait analysis	Walking velocity, stride length, walking frequency, stance phase duration, double stance phase duration
Khazzam	(10)	RA	3D gait analysis	Walking velocity, stride length, cadence, stance duration
Semple	(11)	RA	Electronic Walkway	Walking velocity
Laroche	(12)	RA	3D gait analysis	Walking velocity, walking frequency, stride length, stance duration, double support phase duration
Woodburn	(13)	RA	3D gait analysis	Walking velocity
O'Connell	(14)	RA	Electronic Walkway	Walking velocity, cadence, stride length
Fransen	(15)	RA	Electronic Walkway	Walking velocity, stride length, percentage double support time, stance phase percentage
Zebouni	(16)	AS	Electronic Walkway	Contact time, stride length & stride frequency
Isacson	(17)	RA	Electronic Walkway	Walking velocity, stride length, duration of gait cycle
Minns	(18)	RA	Force sandals	Walking velocity, cadence, stride length, stride width
Simkin	(19)	RA	Force plate	Walking velocity, stride frequency, stride length, stance time, swing time, double support period
Stauffer	(20)	RA	Electronic Walkway	Walking velocity, cadence, stance phase percentage
Del Din	(21)	AS	3D gait analysis	Walking velocity, stride period, stride length, stance period
Mangone	(22)	AS	3D gait analysis	Walking velocity, cadence, stride length
Woodburn	(23)	PsA	Electronic Walkway	Walking velocity
Rome	(24)	Gout	Electronic Walkway	Walking velocity, cadence, step length, stride length, single leg support, double leg support, stance phase duration, swing phase duration

IA = inflammatory arthritis, RA rheumatoid arthritis, AS = Ankylosing spondylitis, PsA = psoriatic arthritis.

Additional File 3: Reference List

1. Dubbeldam R, Nene AV, Buurke JH, Groothuis-Oudshoorn CGM, Baan H, Drossaers-Bakker KW, et al. Foot and ankle joint kinematics in rheumatoid arthritis cannot only be explained by alteration in walking speed. Gait Posture. 2011;33(3):390-5.
2. Rome K, Dixon J, Gray M, Woodley R. Evaluation of static and dynamic postural stability in established rheumatoid arthritis: exploratory study. Clin Biomech. 2009;24(6):524-6.
3. Eppeland S, Myklebust G, Hodt-Billington C, Moe-Nilssen R. Gait patterns in subjects with rheumatoid arthritis cannot be explained by reduced speed alone. Gait Posture. 2009;29(3):499-503.
4. Turner D, Woodburn J. Characterising the clinical and biomechanical features of severely deformed feet in rheumatoid arthritis. Gait Posture. 2008;28(4):574-80.
5. Turner DE, Helliwell PS, Siegel KL, Woodburn J. Biomechanics of the foot in rheumatoid arthritis: Identifying abnormal function and the factors associated with localised disease ‘impact’. Clin Biomech. 2008;23(1):93-100.
6. Weiss RJ, Wretenberg P, Stark A, Palmblad K, Larsson P, Gröndal L, et al. Gait pattern in rheumatoid arthritis. Gait Posture. 2008;28(2):229-34.
7. Turner DE, Helliwell PS, Emery P, Woodburn J. The impact of rheumatoid arthritis on foot function in the early stages of disease: a clinical case series. BMC Musculoskelet Disord. 2006;7:102-8.
8. Turner D, Woodburn J, Helliwell P, Cornwall M, Emery P. Pes planovalgus in RA: a descriptive and analytical study of foot function determined by gait analysis. Musculoskeletal Care. 2003;1(1):21-33.
9. Laroche D, Ornetti P, Thomas E, Ballay Y, Maillefert JF, Pozzo T. Kinematic adaptation of locomotor pattern in rheumatoid arthritis patients with forefoot impairment. Exp Brain Res. 2007;176(1):85-97.
10. Khazzam M, Long JT, Marks RM, Harris GF. Kinematic changes of the foot and ankle in patients with systemic rheumatoid arthritis and forefoot deformity. J Orthop Res. 2007;25(3):319-29.
11. Semple R, Turner DE, Helliwell PS, Woodburn J. Regionalised centre of pressure analysis in patients with rheumatoid arthritis. Clin Biomech. 2007;22(1):127-9.
12. Laroche D, Pozzo T, Ornetti P, Tavernier C, Maillefert JF. Effects of loss of metatarsophalangeal joint mobility on gait in rheumatoid arthritis patients. Rheumatology (Oxford). 2006;45(4):435-40.
13. Woodburn J, Nelson K, Siegel K, Kepple T, Gerber L. Multisegment foot motion during gait: proof of concept in rheumatoid arthritis. J Rheumatol. 2004;31(10):1918-27.
14. O'Connell PG, Lohmann Siegel K, Kepple TM, Stanhope SJ, Gerber LH. Forefoot deformity, pain, and mobility in rheumatoid and nonarthritic subjects. J Rheumatol. 1998;25(9):1681-6.
15. Fransen M, Heussler J, Margiotta E, Edmonds J. Quantitative gait analysis -- comparison of rheumatoid arthritic and non-arthritic subjects. Aust J Physiother. 1994;40(3):191-9.
16. Zebouni L, Helliwell P, Howe A, Wright V. Gait analysis in ankylosing spondylitis. Ann Rheum Dis. 1992;51(7):898-9.
17. Isacson J, Broström LA. Gait in rheumatoid arthritis: an electrogoniometric investigation. J Biomech. 1988;21(6):451-7.
18. Minns R, Craxford AD. Pressure Under the Forefoot in Rheumatoid Arthritis A Comparison of Static and Dynamic Methods of Assessment. Clin Orthop Relat Res. 1984;187:235.
19. Simkin A. The dynamic vertical force distribution during level walking under normal and rheumatic feet. Rheumatol Rehabil. 1981;20(2):88-97.
20. Stauffer RN, Chao EY, Györy AN. Biomechanical gait analysis of the diseased knee joint. Clin Orthop Relat Res. 1977(126):246-55.
21. Del Din S, Carraro E, Sawacha Z, Guiotto A, Bonaldo L, Masiero S, et al. Impaired gait in ankylosing spondylitis. Med Biol Eng Comput. 2011;49(7):801-9.
22. Mangone M, Scettri P, Paoloni M, Procaccianti R, Spadaro A, Santilli V. Pelvis-shoulder coordination during level walking in patients with ankylosing spondylitis. Gait Posture. 2011;34(1):1-5.
23. Woodburn J, Hyslop E, Barn R, McInnes I, Turner D. Achilles tendon biomechanics in psoriatic arthritis patients with ultrasound proven enthesitis. Scand J Rheumatol. 2013(00):1-4.
24. Rome K, Survepalli D, Sanders A, Lobo M, McQueen FM, McNair P, et al. Functional and biomechanical characteristics of foot disease in chronic gout: a case-control study. Clin Biomech. 2011;26(1):90-4.
