# Supplementary material for: Gait characteristics associated with the foot and ankle in inflammatory arthritis: a systematic review and meta-analysis
Source: BMC Musculoskelet Disord. 2015 Jun 5;16:134. doi: 10.1186/s12891-015-0596-0 (PMC4455329; doi:10.1186/s12891-015-0596-0)
Supplement: Additional file 4: — Table S4. Kinematic gait parameters measured and methods of data acquisition. Details of the specific kinematic parameters, measured the biomechanical model used to determine kinematic parameters. [file 12891_2015_596_MOESM4_ESM.pdf]

Gait characteristics associated with the foot and ankle in inflammatory arthritis: a systematic review and meta-analysis

Matthew Carroll, Priya Parmar, Nicola Dalbeth, Mark Boocock, Keith Rome

Additional File 4: Kinematic gait parameters measured and methods of data acquisition

Author	IA	Parameters measured	Kinematic Assessment Method & model	Biomechanical Model
Barn (1)	RA	Peak rearfoot eversion, peak rearfoot plantarflexion, lowest navicular height, peak midfoot inversion, peak forefoot abduction, peak forefoot dorsiflexion	3D analysis	Hyslop (2)
Dubbeldam (2)	RA	Tibio-talar dorsiflexion, medial arch collapse, hallux dorsiflexion, subtalar eversion, mid-/hindfoot supination, fore-/midfoot supination, leg/hindfoot external rotation, forefoot/ankle abduction metatarsal 1-5 angle, hallux abduction	3D analysis	(Heidelberg) Simon (3)
Turner (4)	RA	Rearfoot terminal stance plantarflexion, rearfoot midstance eversion, forefoot midstance inversion, forefoot peak abduction, lowest navicular height, peak hallux dorsiflexion	3D analysis	Carson (5)
Turner (6)	RA	Initial foot contact angle, terminal stance heel rise, minimum navicular height in stance, maximum rearfoot eversion in stance,	3D analysis	Carson (5)
Turner (7)	RA	Initial foot contact angle, terminal stance heel rise, minimum arch height, peak eversion	3D analysis	Carson (5)
Weiss (8)	RA	Trunk tilt range, trunk lateral sway range, hip flexion extension range, hip abduction, knee flexion extension range, ankle plantarflexion, ankle dorsiflexion	3D analysis	Newington (9)
Khazzam (10)	RA	Sagittal, coronal and transverse motion of the hindfoot, forefoot and hallux at load response, midstance, terminal stance, pre-swing, initial swing, mid-swing and terminal swing	3D analysis	Milwaukee (11)
Laroche (12)	RA	Range of motion of MTP joints, foot angle at toe-off	3D analysis	Courtine (13) & Borghese (14)
Laroche (15)	RA	Mean articular amplitudes of MTP joints, mean plantar/dorsi flexion ROM of MTP joints	3D analysis	Courtine (13) & Borghese (14)
Woodburn (16)	RA	Peak dorsiflexion & plantarflexion & ROM, Peak inversion & eversion & ROM, peak adduction, abduction & ROM for the rearfoot & forefoot, minimum & maximum height & displacement of the navicular, peak extension, flexion & ROM of the hallux	3D analysis	Carson (5)
Turner (17)	RA	Max ankle joint dorsi/plantarflexion, maximum inversion/eversion, maximum internal/external rotation, range of ankle joint motion in sagittal, frontal and transvers plane, toe off angle, time to maximum eversion motion time integral of sagittal, frontal and transverse ankle motion	Electromagnetic tracking	N/A
Woodburn (18)	RA	Dorsi/plantarflexion, inversion/eversion & internal/external rotation of ankle joint during stance phase of gait	Electromagnetic tracking	N/A
Woodburn (19)	RA	Dorsi/plantarflexion, inversion/eversion & internal/external rotation of ankle joint during stance phase of gait	Electromagnetic tracking	N/A
O’Connell (20)	RA	Ankle motion during stance, mean heel rise during stance phase of gait	3D analysis	Siegel (21)

Author	IA	Parameters measured	Kinematic Assessment Method & model	Biomechanical Model
Siegel (21)	RA	Foot to floor contact angle, degree of heel rise at toe off, foot out toe angle, plantar/dorsiflexion/inversion/eversion/abduction/adduction of the foot	3D analysis	Siegel (21)
Isacson (22)	RA	Hip, knee & ankle joint flexion/extension, abduction/adduction, rotation	Electrogoniometry	N/A
Stauffer (23)	RA	Standing knee flexion, sagittal, coronal & transverse knee motion, stance phase knee flexion	Electrogoniometry	N/A
Del Din (24)	AS	Flexion/extension, abduction/adduction and internal/external rotation of the trunk, pelvis, hip and ankle. Only flexion/extension reported at the knee	3D analysis	Leardini (25) & Sawacha (26)
Mangone (27)	AS	Pelvis ROM, pelvic tilt, pelvic rotation, shoulder rotation, hip flexion	3D analysis	Davis (28)
Zebouni (29)	AS	Hip flexion & extension, knee flexion & extension	Electrogoniometry	
Woodburn (30)	PsA	Peak ankle/rearfoot dorsiflexion, peak ankle/rearfoot eversion, peak ankle/rearfoot internal rotation, navicular height	3D analysis	Hyslop (31)

IA = inflammatory arthritis, RA rheumatoid arthritis, AS = Ankylosing spondylitis, PsA = psoriatic arthritis, ROM = range of motion, 3D = three-dimensional, N/A = not applicable

Additional File 4: Reference List

1. Barn R, Turner DE, Rafferty D, Sturrock RD, Woodburn J. Tibialis Posterior Tenosynovitis and Associated Pes Plano Valgus in Rheumatoid Arthritis: Electromyography, Multisegment Foot Kinematics, and Ultrasound Features. Arthritis Care & Res. 2013;65(4):495-502.
2. Dubbeldam R, Nene AV, Buurke JH, Groothuis-Oudshoorn CGM, Baan H, Drossaers-Bakker KW, et al. Foot and ankle joint kinematics in rheumatoid arthritis cannot only be explained by alteration in walking speed. Gait Posture. 2011;33(3):390-5.
3. Simon J, Doederlein L, McIntosh A, Metaxiotis D, Bock H, Wolf S. The Heidelberg foot measurement method: development, description and assessment. Gait Posture. 2006;23(4):411-24.
4. Turner D, Woodburn J. Characterising the clinical and biomechanical features of severely deformed feet in rheumatoid arthritis. Gait Posture. 2008;28(4):574-80.
5. Carson M, Harrington M, Thompson N, O'connor J, Theologis T. Kinematic analysis of a multi-segment foot model for research and clinical applications: a repeatability analysis. J Biomech. 2001;34(10):1299-307.
6. Turner DE, Helliwell PS, Siegel KL, Woodburn J. Biomechanics of the foot in rheumatoid arthritis: Identifying abnormal function and the factors associated with localised disease 'impact'. Clin Biomech. 2008;23(1):93-100.
7. Turner DE, Helliwell PS, Emery P, Woodburn J. The impact of rheumatoid arthritis on foot function in the early stages of disease: a clinical case series. BMC Musculoskelet Disord. 2006;7:102-8.
8. Weiss RJ, Wretenberg P, Stark A, Palmblad K, Larsson P, Gröndal L, et al. Gait pattern in rheumatoid arthritis. Gait Posture. 2008;28(2):229-34.
9. Ounpuu S, Gage J, Davis R. Three-dimensional lower extremity joint kinetics in normal pediatric gait. J Pediatr Orthop. 1991;11(3):341.
10. Khazzam M, Long JT, Marks RM, Harris GF. Kinematic changes of the foot and ankle in patients with systemic rheumatoid arthritis and forefoot deformity. J Orthop Res. 2007;25(3):319-29.
11. Myers KA, Wang M, Marks RM, Harris GF. Validation of a multisegment foot and ankle kinematic model for pediatric gait. IEEE Trans Biomed Eng. 2004;12(1):122-30.
12. Laroche D, Ornetti P, Thomas E, Ballay Y, Maillefert JF, Pozzo T. Kinematic adaptation of locomotor pattern in rheumatoid arthritis patients with forefoot impairment. Exp Brain Res. 2007;176(1):85-97.
13. Courtine G, Schieppati M. Human walking along a curved path. I. Body trajectory, segment orientation and the effect of vision. Eur J Neurosci. 2003;18(1):177-90.
14. Borghese NA, Bianchi L, Lacquaniti F. Kinematic determinants of human locomotion. J Physiol. 1996 August 1, 1996;494(Pt 3):863-79.
15. Laroche D, Pozzo T, Ornetti P, Tavernier C, Maillefert JF. Effects of loss of metatarsophalangeal joint mobility on gait in rheumatoid arthritis patients. Rheumatology (Oxford). 2006;45(4):435-40.
16. Woodburn J, Nelson K, Siegel K, Kepple T, Gerber L. Multisegment foot motion during gait: proof of concept in rheumatoid arthritis. J Rheumatol. 2004;31(10):1918-27.
17. Turner D, Woodburn J, Helliwell P, Cornwall M, Emery P. Pes planovalgus in RA: a descriptive and analytical study of foot function determined by gait analysis. Musculoskeletal Care. 2003;1(1):21-33.
18. Woodburn J, Helliwell P, Barker S. Three-dimensional kinematics at the ankle joint complex in rheumatoid arthritis patients with painful valgus deformity of the rearfoot. Rheumatology (Oxford). 2002;41(12):1406-12.
19. Woodburn J, Turner D, Helliwell P, Barker S. A preliminary study determining the feasibility of electromagnetic tracking for kinematics at the ankle joint complex. Rheumatology (Oxford). 1999;38(12):1260-8.

20. Minns R, Craxford AD. Pressure Under the Forefoot in Rheumatoid Arthritis A Comparison of Static and Dynamic Methods of Assessment. Clin Orthop Relat Res. 1984;187:235.
21. Siegel KL, Kepple TM, O'Connell PG, Gerber LH, Stanhope SJ. A technique to evaluate foot function during the stance phase of gait. Foot Ankle Int. 1995;16(12):764-70.
22. Isacson J, Broström LÅ. Gait in rheumatoid arthritis: an electrogoniometric investigation. J Biomech. 1988;21(6):451-3, 5-7.
23. Stauffer RN, Chao EY, Györy AN. Biomechanical gait analysis of the diseased knee joint. Clin Orthop Relat Res. 1977(126):246-55.
24. Del Din S, Carraro E, Sawacha Z, Guiotto A, Bonaldo L, Masiero S, et al. Impaired gait in ankylosing spondylitis. Med Biol Eng Comput. 2011;49(7):801-9.
25. Leardini A, Sawacha Z, Paolini G, Ingrosso S, Nativo R, Benedetti MG. A new anatomically based protocol for gait analysis in children. Gait Posture. 2007;26(4):560-71.
26. Sawacha Z, Gabriella G, Cristoferi G, Guiotto A, Avogaro A, Cobelli C. Diabetic gait and posture abnormalities: A biomechanical investigation through three dimensional gait analysis. Clin Biomech. 2009;24(9):722-8.
27. Mangone M, Scettri P, Paoloni M, Procaccianti R, Spadaro A, Santilli V. Pelvis-shoulder coordination during level walking in patients with ankylosing spondylitis. Gait Posture. 2011;34(1):1-5.
28. Davis RB, Ounpuu S, Tyburski D, Gage JR. A gait analysis data collection and reduction technique. Hum Mov Sci. 1991;10(5):575-87.
29. Zebouni L, Helliwell P, Howe A, Wright V. Gait analysis in ankylosing spondylitis. Ann Rheum Dis. 1992;51(7):898-9.
30. Woodburn J, Hyslop E, Barn R, McInnes I, Turner D. Achilles tendon biomechanics in psoriatic arthritis patients with ultrasound proven enthesitis. Scand J Rheumatol. 2013(00):1-4.
31. Hyslop E, Woodburn J, McInnes I, Semple R, Newcombe L, Hendry G, et al. A reliability study of biomechanical foot function in psoriatic arthritis based on a novel multi-segmented foot model. Gait Posture. 2010;32(4):619-26.
