# Supplementary material for: Gait characteristics associated with the foot and ankle in inflammatory arthritis: a systematic review and meta-analysis
Source: BMC Musculoskelet Disord. 2015 Jun 5;16:134. doi: 10.1186/s12891-015-0596-0 (PMC4455329; doi:10.1186/s12891-015-0596-0)
Supplement: Additional file 5: — Table S5. Kinetic gait analysis parameters acquired by included studies. Details of kinetic gait parameters measured. [file 12891_2015_596_MOESM5_ESM.pdf]

Gait characteristics associated with the foot and ankle in inflammatory arthritis: a systematic review and meta-analysis

Matthew Carroll, Priya Parmar, Nicola Dalbeth, Mark Boocock, Keith Rome

Additional File 5: Kinetic gait parameters measured

Author	Condition	Parameters measured
Barn (1)	RA	Peak ankle power, peak ankle moment
Turner (2)	RA	Peak ankle plantarflexion moment, peak ankle power
Weiss (3)	RA	Joint moments at ankle joint
Turner (4)	RA	Ground reaction forces; peak plantarflexion moment, peak ankle joint power
O'Connell (5)	RA	Ground reaction forces, mean ankle plantarflexion net muscular moment
Siegel (6)	RA	Muscular moment at ankle joint, ground reaction forces
Simkin (7)	RA	Peak force on heel, peak force on midfoot, peak force on 1, 2 metatarsal heads, peak force on 3, 4, 5 metatarsal heads, peak force on toes
Stauffer (8)	RA	Ground reaction forces
Del Din (9)	AS	Flexion-extension, abduction-adduction & internal-external rotation moments at ankle joint
Woodburn (10)	PsA	Peak ankle joint moment, peak ankle joint power, peak Achilles tendon force

RA = rheumatoid arthritis, AS = ankylosing spondylitis, PsA = psoriatic arthritis

Additional File 5: Reference List

1. Barn R, Turner DE, Rafferty D, Sturrock RD, Woodburn J. Tibialis Posterior Tenosynovitis and Associated Pes Plano Valgus in Rheumatoid Arthritis: Electromyography, Multisegment Foot Kinematics, and Ultrasound Features. Arthritis Care & Res. 2013;65(4):495-502.
2. Turner DE, Helliwell PS, Siegel KL, Woodburn J. Biomechanics of the foot in rheumatoid arthritis: Identifying abnormal function and the factors associated with localised disease 'impact'. Clin Biomech. 2008;23(1):93-100.
3. Weiss RJ, Wretenberg P, Stark A, Palmblad K, Larsson P, Gröndal L, et al. Gait pattern in rheumatoid arthritis. Gait Posture. 2008;28(2):229-34.
4. Turner DE, Helliwell PS, Emery P, Woodburn J. The impact of rheumatoid arthritis on foot function in the early stages of disease: a clinical case series. BMC Musculoskelet Disord. 2006;7:102-8.
5. Minns R, Craxford AD. Pressure Under the Forefoot in Rheumatoid Arthritis A Comparison of Static and Dynamic Methods of Assessment. Clin Orthop Relat Res. 1984;187:235.
6. Siegel KL, Kepple TM, O'Connell PG, Gerber LH, Stanhope SJ. A technique to evaluate foot function during the stance phase of gait. Foot Ankle Int. 1995;16(12):764-70.
7. Simkin A. The dynamic vertical force distribution during level walking under normal and rheumatic feet. Rheumatol Rehabil. 1981;20(2):88-97.
8. Stauffer RN, Chao EY, Györy AN. Biomechanical gait analysis of the diseased knee joint. Clin Orthop Relat Res. 1977(126):246-55.
9. Del Din S, Carraro E, Sawacha Z, Guiotto A, Bonaldo L, Masiero S, et al. Impaired gait in ankylosing spondylitis. Med Biol Eng Comput. 2011;49(7):801-9.
10. Woodburn J, Hyslop E, Barn R, McInnes I, Turner D. Achilles tendon biomechanics in psoriatic arthritis patients with ultrasound proven enthesitis. Scand J Rheumatol. 2013(00):1-4.
