# Supplementary material for: Gait characteristics associated with the foot and ankle in inflammatory arthritis: a systematic review and meta-analysis
Source: BMC Musculoskelet Disord. 2015 Jun 5;16:134. doi: 10.1186/s12891-015-0596-0 (PMC4455329; doi:10.1186/s12891-015-0596-0)
Supplement: Additional file 6: — Table S6. Plantar pressure parameters acquired by included studies. Details of the specific regions of the foot where plantar pressure was measured and the method by which plantar pressure was acquired. [file 12891_2015_596_MOESM6_ESM.pdf]

Gait characteristics associated with the foot and ankle in inflammatory arthritis: a systematic review and meta-analysis

Matthew Carroll, Priya Parmar, Nicola Dalbeth, Mark Boocock, Keith Rome

Additional File 6: Plantar pressure gait parameters measured and methods of data acquisition

Author	Condition	Acquisition system	Parameters measured
Bowen (1)	RA	In shoe pressure system	Peak plantar pressure forefoot
Yavuz (2)	RA	Custom device	Peak plantar pressure (1st metatarsal head, hallux, lesser digits); Pressure Time Integral (1st metatarsal head, hallux)
Schmiegel (3)	RA	Plantar pressure plate	Peak plantar pressure (hindfoot, midfoot, metatarsal 1, 2, 3-5, hallux, 2nd toe, toes 3-5)
Schmiegel (4)	RA	Plantar pressure plate	Peak plantar pressure (hindfoot, midfoot, metatarsal 1, 2, 3-5, hallux, 2nd toe, toes 3-5)
Turner (5)	RA	Plantar pressure plate	Centre of pressure at 50% of foot length, lesser toe contact area, midfoot contact area, forefoot peak pressure
Turner (6)	RA	Plantar pressure plate	Centre of pressure at 50% of foot length, lesser toe contact area, midfoot contact area, forefoot peak pressure
Semple (7)	RA	Plantar pressure plate	Velocity and duration centre of pressure
Rosenbaum (8)	RA	Plantar pressure plate	Peak plantar pressure (hindfoot, midfoot, metatarsal 1, 2, 3-5, hallux, 2nd toe, toes 3-5)
Turner (9)	RA	Plantar pressure plate	Centre of pressure at 50% of foot length, lesser toe contact area, midfoot contact area, forefoot peak pressure
Tuna (10)	RA	Plantar pressure plate	Peak plantar pressure (hindfoot, midfoot, forefoot phalanx)
Otter (11)	RA	Plantar pressure plate	Peak plantar pressure (forefoot), force time integral (forefoot)
Turner (12)	RA	Plantar pressure plate	Peak plantar pressure and force, PTI & FTI, total contact area for hindfoot, midfoot forefoot, hallux & digits
O'Connell (13)	RA	Force plate system	Centre of pressure at 50% of foot length
Woodburn (14)	RA	In shoe pressure system	Peak plantar pressure (metatarsal heads 1-5)
Minns (15)	RA	Plantar pressure plate	NR
Rome (16)	Gout	In shoe pressure system	Peak plantar pressure (hindfoot, midfoot, 1st , 2nd , 3rd, 4th & 5th metatarsal heads, hallux, toes 2-5

RA = rheumatoid arthritis, PTI = pressure time integral, FTI = force time integral, NR = not reported

Additional File 6: Reference List

1. Bowen CJ, Culliford D, Allen R, Beacroft J, Gay A, Hooper L, et al. Forefoot pathology in rheumatoid arthritis identified with ultrasound may not localise to areas of highest pressure: cohort observations at baseline and twelve months. J Foot Ankle Res. 2011;4(1):25-.
2. Yavuz M, Husni E, Botek G, Davis BL. Plantar Shear Stress Distribution in Patients with Rheumatoid Arthritis Relevance to Foot Pain. J Am Podiatr Med Assoc. 2010;100(4):265-9.
3. Schmiegel A, Rosenbaum D, Schorat A, Hilker A, Gaubitz M. Assessment of foot impairment in rheumatoid arthritis patients by dynamic pedobarography. Gait Posture. 2008;27(1):110-4.
4. Schmiegel A, Vieth V, Gaubitz M, Rosenbaum D. Pedography and radiographic imaging for the detection of foot deformities in rheumatoid arthritis. Clin Biomech. 2008;23(5):648-52.
5. Turner D, Woodburn J. Characterising the clinical and biomechanical features of severely deformed feet in rheumatoid arthritis. Gait Posture. 2008;28(4):574-80.
6. Turner DE, Helliwell PS, Siegel KL, Woodburn J. Biomechanics of the foot in rheumatoid arthritis: Identifying abnormal function and the factors associated with localised disease 'impact'. Clin Biomech. 2008;23(1):93-100.
7. Semple R, Turner DE, Helliwell PS, Woodburn J. Regionalised centre of pressure analysis in patients with rheumatoid arthritis. Clin Biomech. 2007;22(1):127-9.
8. Rosenbaum D, Schmiegel A, Meermeier M, Gaubitz M. Plantar sensitivity, foot loading and walking pain in rheumatoid arthritis. Rheumatology (Oxford). 2006;45(2):212-4.
9. Turner DE, Helliwell PS, Emery P, Woodburn J. The impact of rheumatoid arthritis on foot function in the early stages of disease: a clinical case series. BMC Musculoskelet Disord. 2006;7:102-8.
10. Tuna H, Birtane M, Taştekin N, Kokino S. Pedobarography and its relation to radiologic erosion scores in rheumatoid arthritis. Rheumatol Int. 2005;26(1):42-7.
11. Otter SJ, Bowen CJ, Young AK. Forefoot plantar pressures in rheumatoid arthritis. J Am Podiatr Med Assoc. 2004;94(3):255-60.
12. Turner D, Woodburn J, Helliwell P, Cornwall M, Emery P. Pes planovalgus in RA: a descriptive and analytical study of foot function determined by gait analysis. Musculoskeletal Care. 2003;1(1):21-33.
13. O'Connell PG, Lohmann Siegel K, Kepple TM, Stanhope SJ, Gerber LH. Forefoot deformity, pain, and mobility in rheumatoid and nonarthritic subjects. J Rheumatol. 1998;25(9):1681-6.
14. Woodburn J, Helliwell PS. Relation between heel position and the distribution of forefoot plantar pressures and skin callosities in rheumatoid arthritis. Ann Rheum Dis. 1996;55(11):806-10.
15. Minns R, Craxford AD. Pressure Under the Forefoot in Rheumatoid Arthritis A Comparison of Static and Dynamic Methods of Assessment. Clin Orthop Relat Res. 1984;187:235.
16. Rome K, Survepalli D, Sanders A, Lobo M, McQueen F, McNair P, et al. Functional and biomechanical characteristics of foot disease in chronic gout: A case-control study. Clin Biomech. 2011;26(1):90-4.
